# Cu_3_P/RGO Nanocomposite as a New Anode for Lithium-Ion Batteries

**DOI:** 10.1038/srep35189

**Published:** 2016-10-11

**Authors:** Shuling Liu, Xiaodong He, Jianping Zhu, Liqiang Xu, Jianbo Tong

**Affiliations:** 1 College of Chemistry & Chemical Engineering, Shaanxi University of Science & Technology, Xi’an Shaanxi, 710021, P. R. China; 2 School of Chemistry and Chemical Engineering, Shandong University, Ji’nan Shandong, 250100, P. R. China

## Abstract

Cu_3_P/reduced graphene oxide (Cu_3_P/RGO) nanocomposite was successfully synthesized by a facile one-pot method as an advanced anode material for high-performance lithium-ion batteries. Cu_3_P nanostructures with a polyhedral shape with the mean diameter (80–100 nm) were homogeneously anchored on the surface of RGO. The flexible RGO sheets acted as elastic buffering layer which not only reduced the volume change, but also prevented the aggregation of Cu_3_P nanostructures, the cracking and crumbing of electrodes. On the other hand, the presence of Cu_3_P nanostructures could also avoid the agglomeration of RGO sheets and retain their highly active surface area. Therefore, as an advanced anode material for high-performance lithium-ion batteries, the as-prepared Cu_3_P/RGO exhibited high capacity of 756.15 mAhg^−1^ at the current density 500 mAg^−1^ after 80 cycles, superior cyclic stability and good rate capability.

Lithium-ion rechargeable batteries (LIBs) have currently become the research focus on the increasing demand for portable electronic devices (e.g., mobile phones and laptops) in our daily life. However, currently used anode material such as commercial graphite exhibits a relatively low theoretical capacity of 372 mAhg^−1^, which leads to a limited energy output of LIBs^1^. To meet the ever-growing performance requirements for practical application, many efforts have been spent on seeking alternative anode materials. To date, some new anode materials have also been reported, such as metal oxides^2^,^3^, non metal^4^, and metal phosphides^5^,^6^,^7^,^8^.

Among these anode materials, transition metal phosphides (M-P, where M = Fe, Co, Ni, Cu, etc.) have attracted increasing attention owing to their high gravimetric and volumetric capacity associated with the low polarization and good cycling stability^9^. Copper phosphide is one of the most important transition metal phosphides, and its gravimetric capacity is closed to that of graphite. Its volumetric capacity is almost three times higher than that of graphite (3020 mA h·cm^−3^ for Cu_3_P and 830 mA h·cm^−3^ for graphite). However, similar to transition metal oxides, it usually suffers from rapid capacity fading, limited cycling life, and poor high-rate performance because of the intrinsic volume effect^10^,^11^,^12^ and poor conductivity during the lithium insertion/exaction process. To address the problem, a variety of appealing strategies have been utilized to alleviate these intractable problems. Size miniaturization and control of morphology had proved to effectively enhance the electrochemical performance, such as hierarchical dendrites^13^,^14^, and hexagonal plate-like^15^,^16^. Additionally, combining highly conductive carbon materials is also beneficial to enhance average electronic conductivity of active material, providing conductive electronic wiring between the active particles and current collector^17^. Graphene is the most popular and intriguing two-dimensional carbon material due to its superior electrical conductivity, large surface area, chemical stability, and structural flexibility^18^,^19^,^20^,^21^. More importantly, graphene can also be used in nanocomposites with transition metal phosphide nanoparticles (NPs) to improve the electrochemical performance of these particles^17^. The graphene could not only provide support for anchoring well-dispersed NPs, increasing conductivity and surface area of the electrodes, but also can effectively prevent the volume expansion/contraction and aggregation of NPs during Li charge/discharge process^22^. Meanwhile, the anchoring of NPs on graphene can effectively reduce the degree of restacking of graphene^23^ sheets and consequently keep their highly active surface area, and increase lithium storage capacity and cyclic performance of graphene-based material to some extent. Therefore, it is expected that the anchoring of Cu_3_P nanostructure on graphene can efficiently improve the electrochemical activity of LIBs and obtain the nanocomposite with superior lithium storage properties.

Herein, we report a facile strategy to synthesize Cu_3_P/RGO nanocomposite as an advanced anode material for high performance LIBs. The as-prepared Cu_3_P/RGO nanocomposite (Cu_3_P nanostructures in polyhedral shape homogeneously anchored on the RGO sheets) has a high capacity of 756.15 mAhg^−1^ at the current density 500 mAg^−1^ after 80 cycles, which is higher than the previous reported 315 mAhg^−1^ of Sn_4_P_3_ after 200 cycles at a rate of 200 mAg^−1^, 224 mAhg^−1^ after 10 cycles of Cu_3_P and 736.8 mAhg^−1^ of CuO NSs/RGO (CuO nanosheets/reduced-graphene oxide nanocomposite) after 50 cycles at rate of 0.1 C (67 mAg^−1^)^24^,^25^,^26^. Superior cyclic stability, and rate capability, which may be a promising electrode material and applied in the energy storage of high-performance lithium-ion batteries.

## Results and Discussion

**Figure 1:**
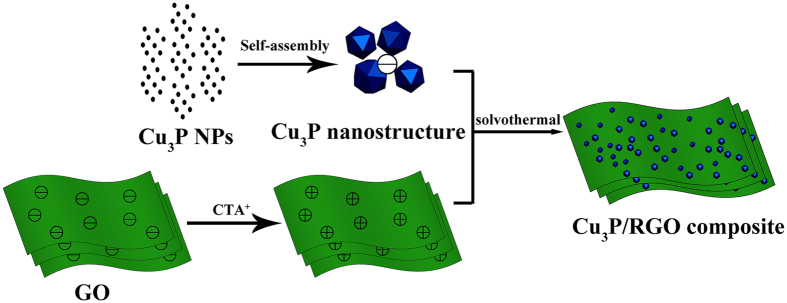
The Schematic layout of the experimental process for the preparation of Cu_3_P/RGO nanocomposite.

Figure 1 describes the experimental procedure of the preparation of Cu_3_P/RGO nanocomposite by a simple solvent method. First of all, the surfaces of the as-prepared graphene oxide (GO) sheets were negatively charged. After cetyltrimethyl ammonium bromide (CTAB) was added, the zeta potential of graphene oxide was modified from negative to positive (see Supplementary Fig. S1). CTAB can also partially reduce the GO nanosheets^27^. Besides, Cu^2+^ could form [Cu(NH_3_)_4_]^2+^ in the alkaline ammonia system and then gradually released the free Cu^2+^ as the reactions were initiated. The released Cu^2+^ would react with PH_3_ (originated from the disproportionation of phosphorus) to produce Cu_3_P nanoparticles (NPs) and then grow into polyhedral nanostructures. Furthermore, the surfaces of these polyhedral nanostructures had negative charges (see Supplementary Fig. S1). Due to the electrostatic interaction, the as-obtained Cu_3_P nanostructures with negative charge were strongly anchored on the modified surface of GO and then formed Cu_3_P/RGO nanocomposites. The nanocomposite had a high structure stability (Cu_3_P nanostructures still anchored on the surface of RGO sheets) even after a long sonication process (in order to disperse the nanocomposite in ethanol for TEM observation), which indicates the strong interaction between Cu_3_P nanostructures and RGO sheets. The whole reactions in the solvothermal system can be described in the following Equations (1–4).


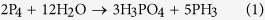



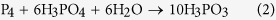



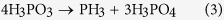



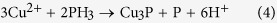


**Figure 2:**
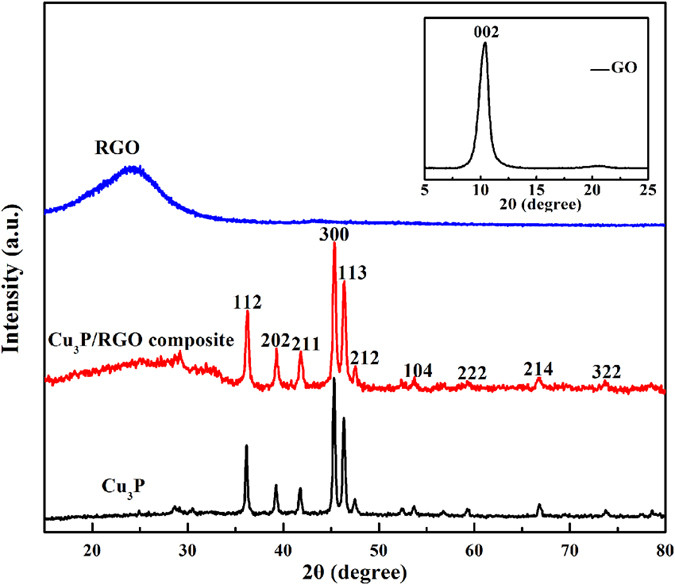
XRD patterns of GO (inset), RGO, Cu_3_P and Cu_3_P/RGO nanocomposite.

XRD patterns were used to characterize the crystal structures of GO, Cu_3_P, RGO and Cu_3_P/RGO nanocomposite, and the results are shown in Fig. 2. The characteristic diffraction peak GO (inset in Fig. 2) at 10.4° corresponds to an interlayer spacing of 0.850 nm^28^, which indicates that GO has been successfully synthesized. After the hydrothermal reaction, this peak disappears following by the presence of a new broad diffraction peak at approximately 24.4° with an interlayer spacing of 0.365 nm^28^, which could be referred to the (002) diffraction of RGO. This could also prove the GO has been reduced to RGO under the hydrothermal treatment. The phase purity of both the as-prepared Cu_3_P nanostructure and Cu_3_P/RGO nanocomposite has also been investigated; all the diffraction peaks can be perfectly assigned to hexagonal Cu_3_P (JCPDS card no. 71–2261) except the weak peak of RGO. The presence of the RGO weak peak indicates that the restacking of RGO nanosheets is prevented by the attached Cu_3_P nanostructures. Supplementary Fig. S2 shows the corresponding EDS microanalysis of the as-prepared Cu_3_P/RGO nanocomposite. The result confirms the coexistence of Cu, P, O, and C in the Cu_3_P/RGO nanocomposite, which is in pretty good agreement with the XRD result. Furthermore compared with 1:1 in GO (from XPS and EDS of Supplementary Figs S3 and S4), the ratio of C and O in the Cu_3_P/RGO product is nearly 11:1, which shows a large number of oxygen-containing functional groups have been removed after chemical reduction. As the RGO sheet has excellent electronic conductivity, it may serve as the conductive channels between Cu_3_P nanostructures and is favorable for stabilizing the electronic and ionic conductivity as a result^29^.

**Figure 3:**
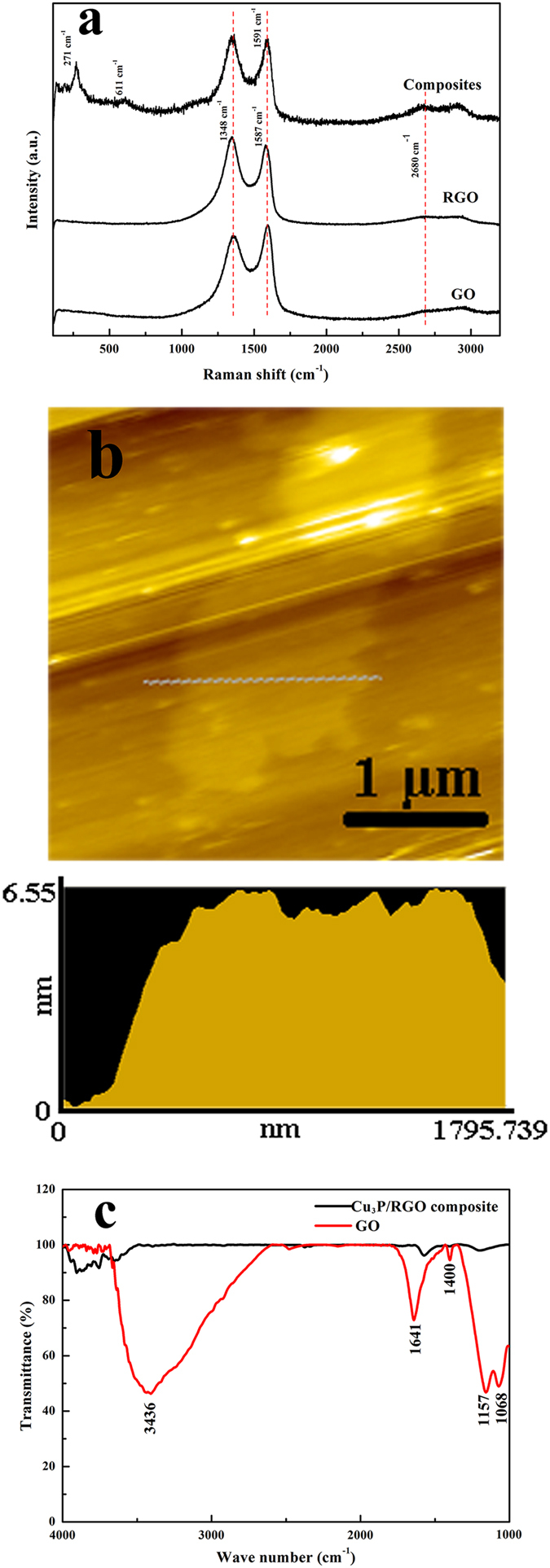
(**a**) Room-temperature Raman spectra of Cu_3_P/RGO nanocomposite, GO and RGO, (**b**) AFM of GO, (**c**) FTIR of Cu_3_P/RGO nanocomposite and GO.

Figure 3 shows the Raman spectra of GO, RGO and Cu_3_P/RGO nanocomposite, respectively. There are two distinct peaks of the carbon material corresponding to two different vibration modes of atoms. The peak at 1350 cm^−1^ for D band indicates the breathing mode of κ-point photon of A_1g_ symmetry while that at 1575 cm^−1^ for G band corresponds to the first order scattering of the E_2g_ phonon of sp^2^ C atoms^30^. In this system, there are two prominent peaks at 1348 cm^−1^ and 1591 cm^−1^ for the Cu_3_P/RGO nanocomposite, which can be accordingly assigned to the D and G bands of RGO^31^. Also among the two bands, the G band shift in carbon-based nanocomposites relates to the charge transfer between the carbon and other compounds present^32^,^33^,^34^. Therefore, the observed shift by 4 cm^−1^ from 1587 cm^−1^ (RGO) to 1591 cm^−1^ (Cu_3_P/RGO nanocomposite) indicates the presence of charge transfer from RGO to Cu_3_P nanostructures. The charge transfer can also be supported by the result of XPS analysis (Supplementary Fig. S3). Meanwhile, it is also found that the Cu_3_P/RGO nanocomposite display relatively higher intensity ratio of D to G band (1.07) than that of GO (0.86). This further confirms that GO is reduced to RGO after the solvothermal process^35^. Except these two peaks, two other Raman peaks located at 271 and 611 cm^−1^ correspond to the typical peaks of Cu_3_P. In addition, the peak at about 2680 cm^−1^ of GO is assigned to 2D brand resulted from two phonon double resonance Raman process. The single RGO and RGO of the composite show a broader peak demonstrating that the present RGO is in few-layer form. Atomic Force Microscope (AFM, Fig. 3b) analysis also confirms that the thickness of graphene oxide is about 6.55 nm and there are about 3-4 layers that belong to the few-layer-graphene oxide. Figure 3c shows Fourier transform infrared spectrum (FTIR) of GO and RGO. GO shows a strong peak at 3436 cm^−1^ related to water O-H stretching vibration. The presence of other oxygen containing functional groups in GO gives rise to major peaks at 1068 cm^−1^ (C-O stretching), 1157 cm^−1^ (epoxy group), 1641 cm^−1^ (skeletal vibrations from unoxidized graphitic domains). But after the solvothermal process, the strong broad peak from O-H becomes weak and shows a blue shift, and other peaks have all disappeared, which further proves GO being reduced which is in agreement with the result of Raman spectra.

**Figure 4:**
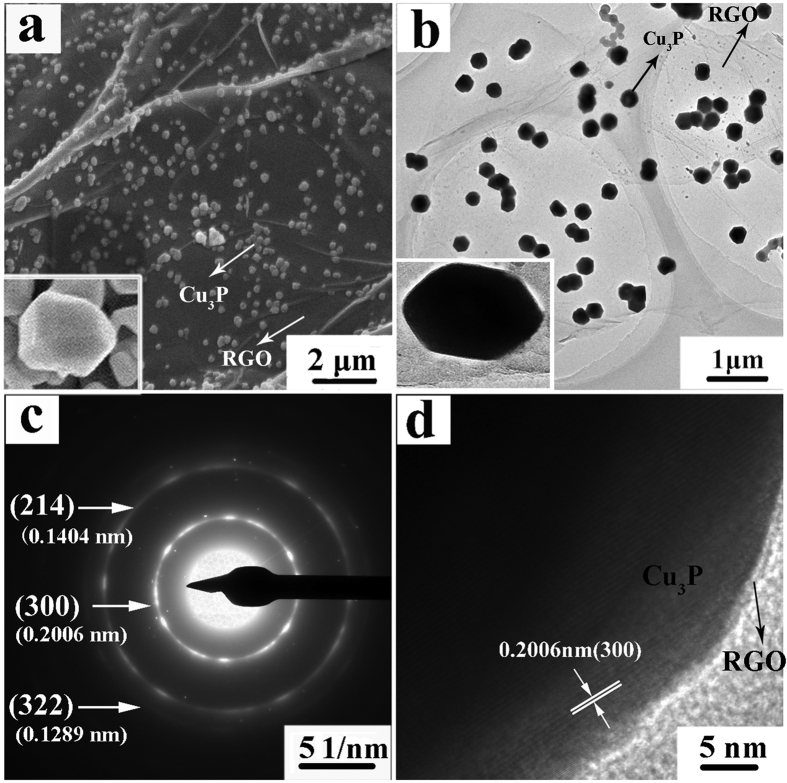
(**a**) FESEM image, (**b**) TEM image, (**c**) SAED pattern, and (**d**) HRTEM image of Cu_3_P/RGO nanocomposite.

The microstructure of the Cu_3_P/RGO nanocomposite was examined by field emission scanning electron microscope (FESEM), transmission electron microscopy (TEM), selected area electron diffraction (SAED) and high-resolution transmission electron microscope (HRTEM) (Fig. 4). FESEM image (see Fig. 4a) illustrates that the RGO sheets are decorated by Cu_3_P nanostructures with the diameters in the range of 80–100 nm. The Cu_3_P nanostructures are evenly and tightly distributed on RGO sheets, and no large aggregation is detected. The inset image in Fig. 4a further proves the polyhedral characteristics of Cu_3_P nanostructures. From the low-magnification TEM image (see Fig. 4b), it can also be seen that all the Cu_3_P nanostructures are anchored on the RGO nanosheets and there are no individual nanostructures. Closer observation (the insert image) reveals these nanostructures are polyhedral with almost no aggregation. The morphology and size of nanostructures are consistent with that of the SEM observation. The corresponding SAED pattern (see Fig. 4c) suggests the polycrystalline nature of Cu_3_P nanostructures in the Cu_3_P/RGO nanocomposite. These ring patterns can be separately assigned to the (300), (214), and (322) reflections of the hexagonal Cu_3_P (JCPDS no. 71–2261), which are consistent with the XRD results. The HRTEM image (see Fig. 4d) displays the high-crystalline of nanostructures. The lattice fringe spacing between two adjacent crystal planes of the nanostructures is determined to be 0.20 nm and can be well indexed as the (300) lattice plane of hexagonal structure Cu_3_P, which right meets the XRD (see Fig. 2) and SAED results (see Fig. 4c).

**Figure 5:**
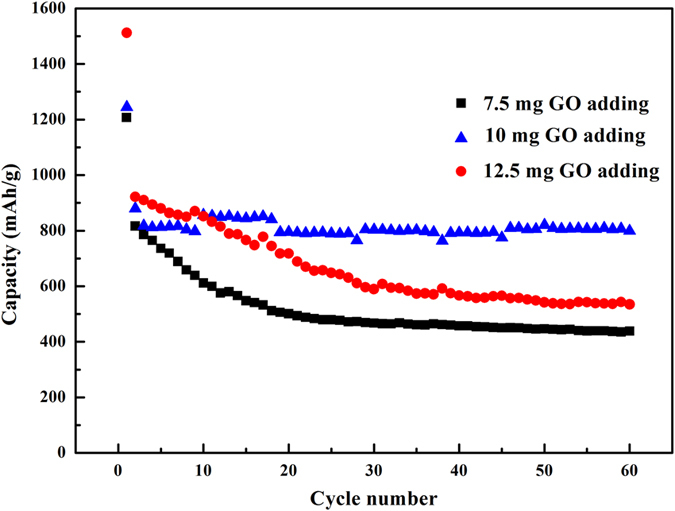
Cycle performance of sample I, sample II, sample III.

**Figure 6:**
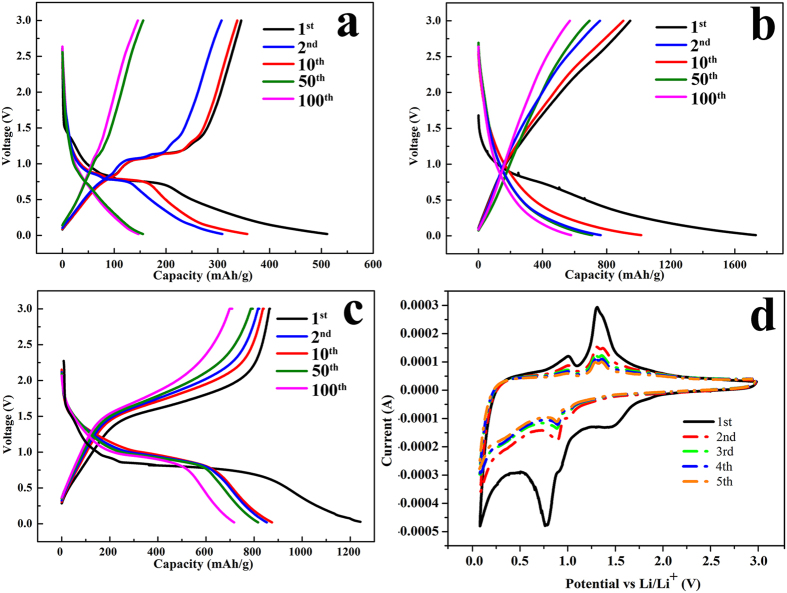
The 1^st^, 2^nd^, 10^th^, 50^th^, 100^th^ charge/discharge curves of (**a**) Cu_3_P, (**b**) RGO and (**c**) Cu_3_P/RGO nanocomposite at a current density of 500 mAg^−1^. (**d**) Cyclic voltammograms of Cu_3_P/RGO nanocomposite at a scanning rate of 0.2 mV/s.

It is well known that the molar ratio of the components in composite can always affect the properties of the as-prepared composite. As a result, three samples with different RGO content was prepared to optimize the product. To be specific, GO was added as follows: 7.5 mg (sample I), 10 mg (sample II) and 12.5 mg (sample III). Figure 5 shows the cycle performance of the three samples. At the current density of 500 mAg^−1^ and after 60 cycles, the reversible capacity of sample I was 438.53 mAhg^−1^ while they were 799.28 mAhg^−1^ for sample II and 534.90 mAhg^−1^ for sample III. After comparison, the above results suggest that the optimized RGO content in the nanocomposite was 10 mg. And then the electrochemical performances of Cu_3_P nanostructures, RGO and Cu_3_P/RGO nanocomposite (sample II) were first evaluated by galvanostatic charge/discharge measurements in the voltage range of 0.01–3.0 V at a current density of 500 mAg^−1^ (see Fig. 6), respectively. As shown in Fig. 6, the first discharge capacities of Cu_3_P, RGO and Cu_3_P/RGO nanocomposite are 511.21, 1730.58 and 1244.69 mAhg^−1^, respectively. It is suggested that the irreversible capacity between the first discharge and charge is mainly due to the solid electrolyte interface (SEI) film which forms during the low potential range^36^. At the second cycle, Cu_3_P/RGO nanocomposite demonstrates much better electrochemical lithium storage performance than Cu_3_P electrode. After ten charge/discharge cycles, it shows a high reversible capacity of about 800 mAhg^−1^. RGO displays a larger first charge-discharge capacity, however, it expresses a low initial coulombic efficiency (about 58.69%) and quick reversible capacity lost after 10th cycle (see Fig. 6b). Similarly, the reversible capacity of Cu_3_P rapidly decreases to 308.58 mAhg^−1^ with a low coulombic efficiency of 93.7% and then increases to 97.3% after 50th cycle (see Fig. 6a). As a comparison, the coulombic efficiency of Cu_3_P/RGO nanocomposite at the first cycle is 70.68% and it rises rapidly to 97.9% in tenth cycle and keeps above 98% in the following cycles (see Fig. 6c), showing that the irreversible loss is diminishing rapidly upon cycling. Moreover, from Fig. 6a,c, the small voltage plateaus for Cu_3_P and Cu_3_P/RGO at 1.30 and 1.5 V can be assigned to the insertion of a small amount of lithium^6^. And their long flat voltage plateaus are also observed at 0.75 and 0.77 V, respectively, which can be attributed to the reaction of Equation (5).


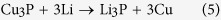


However, the Cu_3_P/RGO nanocomposite has a longer and more stable discharge platform than the single-component Cu_3_P nanostructures and other Cu_3_P morphology such as hollow spheres^25^, indicating that RGO reduces the volume effect (generated by Cu_3_P nanostructures) efficiently. It is evidenced that the excellent electrical properties of RGO improve the conductivity of Cu_3_P/RGO nanocomposite, shortening the distance of lithium-ion transmission and improve the diffusion rate of lithium-ion deintercalation process. No obvious voltage plateau is observed for RGO (see Fig. 6b)^37^.

Figure 6d presents cyclic voltammograms (CV) of the Cu_3_P/RGO nanocomposite electrodes. Two cathodic peaks are observed at 0.77 and 1.5 V in the first cycle, corresponding to a multi-step electrochemical reduction (lithium) reaction of Cu_3_P with Li^38^, which matches well with the voltage plateaus in the charge/discharge profiles. The main anodic peak appeared at 1.25 V is ascribed to the oxidation (delithiation) reaction of Cu_3_P. The formation of Cu and Li_3_P and the re-formation of Cu_3_P can be described by the Equation (6).


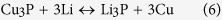


The main reduction peak is shifted to 0.90 V after the initial cycle, indicating the effect of SEI film formed during the first cycle, the intensity and integral areas of the peak of the third cycle are close to that of the fourth one. This consequence indicates that the electrochemical reversibility of Cu_3_P/RGO is gradually built after the second cycle.

**Figure 7:**
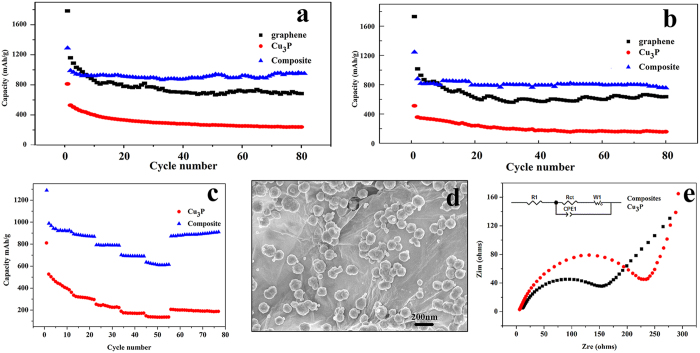
Cycle performance of Cu_3_P, RGO, Cu_3_P/RGO nanocomposite at a current density of (**a**) 100 mAg^−1^, (**b**) 500 mAg^−1^. Rate capability of the Cu_3_P/RGO nanocomposite and Cu_3_P (**c**) at various current densities between 100 and 1600 mAg^−1^. (**d**) SEM of Cu_3_P/RGO nanocomposite after cycling. (**e**) Nyquist plots of Cu_3_P/RGO nanocomposite and Cu_3_P electrodes.

Figure 7a,b show the cycle performance of Cu_3_P, RGO and Cu_3_P/RGO nanocomposite at the current density of 100 and 500 mAg^−1^. Obviously, Cu_3_P/RGO shows a much better cycling performance and stability than Cu_3_P and RGO. At a current density of 500 mAg^−1^, the reversible capacity of Cu_3_P and RGO drop from 356.65 and 1015.72 mAhg^−1^ to 158.36 and 636.47 mAhg^−1^ respectively, corresponding to 44.40% and 62.66% capacity retention based on a second discharge cycle. As a comparison, Cu_3_P/RGO exhibits a high reversible capacity after the initial discharge for 756.15 mAhg^−1^ corresponding to 85.95% capacity retention. This capacity is preferable compared with the values reported previously. (see Table 1) Considering the electrochemical performances of RGO and Cu_3_P, the capacity retention of Cu_3_P/RGO may be related with the interfacial interaction between RGO and Cu_3_P, which can possibly promote the quick transfer of electron between RGO and Cu_3_P and leads to the higher capacity and stability. In addition, the rate performances of the Cu_3_P/RGO nanocomposite and Cu_3_P are also examined by charging/discharging the cells at different current densities from 100 to 1600 mAg^−1^ and back to 100 mAg^−1^ for 11 cycle interval each (see Fig. 7c, Supplementary Table S1). Apparently, although both Cu_3_P/RGO and Cu_3_P electrode restore their original capacity or even a little bit higher when the rate returns to the initial 100 mAg^−1^ after 55 cycles (887.52 mAhg^−1^ for Cu_3_P/RGO and 195.07 mAg^−1^ for Cu_3_P), Cu_3_P/RGO nanocomposite shows much better rate capability than that of the Cu_3_P electrode operated at various rates between 100 and 1600 mAg^−1^, indicating its high reversibility and excellent cyclability. The excellent high-rate performance may be attributed to the unique structure of Cu_3_P/RGO nanocomposite, in which RGO sheets and Cu_3_P nanostructure are directly connected, improving the electrical conductivity of the electrode. But it is known that the morphology can always affect their property of nanomaterials. So the morphology of Cu_3_P nanostructures after cycling was further investigated (see Fig. 7d). It is easy to find that the morphology and size of Cu_3_P nanostructures are almost unchanged, and there is no apparent aggregation, which may be one of reasons of good cycling performance.

To further explore contribution of RGO to the electrochemical performance of Cu_3_P/RGO nanocomposite, the electrochemical impedance spectroscopic analysis for the half cell was carried out (see Fig. 7e). The semicircle at high-medium frequency represents the charge transfer resistance. As shown in Fig. 7e, the Nyquist plots for Cu_3_P/RGO nanocomposite shows smaller semicircle diameters than Cu_3_P, indicating lower charge transfer resistance. The EIS data demonstrates that a good interaction between RGO sheets and Cu_3_P nanostructures in the nanocomposite. RGO’s conductive network significantly facilitate charge transfer and reduce the overall internal resistance of the cell^39^, which accounts for its stable and improved cycling and rate performances. Cu_3_P nanostructures anchor homogeneously on the RGO sheets with a flexible two-dimensional structure. This flexible structure is an elastic buffer space, which not only can accommodate the volume expansion effect of Cu_3_P nanostructures during the Li insertion/extraction, but also can prevent the aggregation of Cu_3_P nanostructures and the cracking and crumbing of electrode^40^,^41^. Instead, the presence of Cu_3_P nanostructures avoids effectively the agglomeration of RGO sheets, which further keeps its high active surface area. The diffusion of Li-ion depends on the transport length and the active site of material^42^. The high active surface area of as-prepared Cu_3_P/RGO nanocomposite can shorten path length for Li^+^ transport and provide more accessible active site for the Li-ion diffusion of the battery reactions, which leads to improved electrochemical performance of the as-prepared Cu_3_P/RGO nanocomposite in LIBs.

## Conclusions

In this report, the Cu_3_P/RGO nanocomposite was synthesized by a one-pot solvothermal method. Cu_3_P with polyhedral nanostructures were found to anchor homogeneously on the surface of RGO sheets. The as-prepared Cu_3_P/RGO nanocomposite exhibits a high initial discharge capacity of 1244.7 mAhg^−1^ at a current density of 500 mAg^−1^, good rate capability and superior cyclic performance and so on, which may be applied in the energy storage of high-performance lithium-ion batteries.

## Methods

### Materials

All the chemical reagents were analytical pure grade and were used without further treatment.

### Synthesis of Cu_3_P/RGO Nanocomposite

The GO in this work was synthesized from the natural graphite power by a modified Hummer’s method as described elsewhere^43^. An appropriate amount of GO (10 mg) was added into 40 ml ammonia liquor (28%) and a homogeneous suspension was obtained after ultrasonication, which was then transferred into a Teflon-lined autoclave of 50 ml capacity. Cetyltrimethyl ammonium bromide (CTAB, 0.05 g) and copper chloride dehydrate (CuCl_2_·2H_2_O, 0.05 g) were added under stirring. After the mixture became a homogeneous suspension, yellow phosphorus (YP, 0.1 g) was added. The autoclave was sealed and maintained at 140 °C for 12 h, then cooled to room temperature naturally. The resulting black precipitate was separated by centrifugation and washed respectively with distilled water, benzene and absolute ethanol. Finally, the as-prepared products were dried in a vacuum at 60 °C for 6 h and collected for characterization for the next step.

### Characterization

The X-ray powder diffraction (XRD) patterns of the as-prepared products were recorded by a Japan Rigaku D/Max-3c X-ray diffraction solutions with a Cu Kα radiation (λ = 1.5418 Å). The crystal structure, surface morphology and particle size of the as-prepared products were examined by field emission scanning electron microscope (FESEM, Hitachi S-4800, Japan) with an energy dispersive spectrometer (EDS), transmission electron microscopy (TEM), high-resolution transmission electron microscope (HRTEM) and selected area electron diffraction (SAED) on a FEI Tecnai G^2^ F20 apparatus with an accelerating voltage of 200 kV. Raman spectra were measured on a LABRAM-HR laser confocal microRaman, spectrometer X-ray photoelectron spectroscopy (XPS) on an AXIS SUPRA (Kratos).

### Electrochemical Testing

The electrochemical response of the samples was performed in two-electrode cells^44^. Working electrodes were prepared by mixing active material (as-prepared sample) with conductivity agent (carbon black) and poly (vinylidene difluoride) in a solvent (N-methyl-2-pyrrolidone) in the weight ratio of 8:1:1. The mixture was pasted on Cu foil as the electrode after ball milling for 2 h. This electrode sheet was dried at 120 °C in vacuum oven for 12 h and then cut into disk shape. The active material of every working electrode is between 1 mg and 1.5 mg. Celgard 2340 was used as a separator and the electrolyte used was 1 M solution of LiPF_6_ in ethylene carbonate (EC)/diethylene carbonate (DEC)/ethyl methyl carbonate (EMC) (1: 1: 1, v/v/v). Li metal foil was used as counter electrode. CR2016 coin cell was assembled in an argon-filled dry box. The galvanostatic charge-discharge and rate capability tests were carried out on LAND CT2001A system. CV measurements were carried out using a Solartron 1287 electrochemical workstation at a scanning rate of 0.2 mV/s. Electrochemical impedance spectroscopy (EIS) measurements were performed on this apparatus from a 1 Hz to 100 kHz frequency range with a 5 mV amplitude.

## Additional Information

**How to cite this article**: Liu, S. *et al*. Cu_3_P/RGO Nanocomposite as a New Anode for Lithium-Ion Batteries. *Sci. Rep.* **6**, 35189; 10.1038/srep35189 (2016).

## Supplementary Material

Supplementary Information

## Figures and Tables

**Table 1:** Comparison of Various materials for LIBs.

materials	Current density (mAg^−1^)	Cycle number	Specific capacity (mAhg^−1^)
Sn_4_P_3_	200	200	315^24^
Ni_2_P/graphene sheet hybrid	54	50	449.9^45^
Cu_3_P	224	10	224^25^
CoP/carbon	179	100	630^46^
SiCN–graphene composite	40	100	475^47^
Cu_3_P/RGO nanocomposite	500	80	756(this work)
