# Supplementary material for: Cu3P/RGO Nanocomposite as a New Anode for Lithium-Ion Batteries
Source: Sci Rep. 2016 Oct 11;6:35189. doi: 10.1038/srep35189 (PMC5057080; doi:10.1038/srep35189)
Supplement: Supplementary Information [file srep35189-s1.pdf]

Supplementary Information

Cu_3P/RGO Nanocomposite as a New Anode for Lithium-Ion Batteries

Shuling Liu[1,*], Xiaodong He[1], Jianping Zhu[1], Liqiang Xu[2], and Jianbo Tong[1]

[1]College of Chemistry & Chemical Engineering, Shaanxi University of Science & Technology, Xi'an Shaanxi, 710021, PR China.

[2]School of Chemistry and Chemical Engineering, Shandong University, Ji'nan Shandong, 250100, PR China

*shulingliu@aliyun.com

* Corresponding author:

Shuling Liu, E-mail: shulingliu@aliyun.com

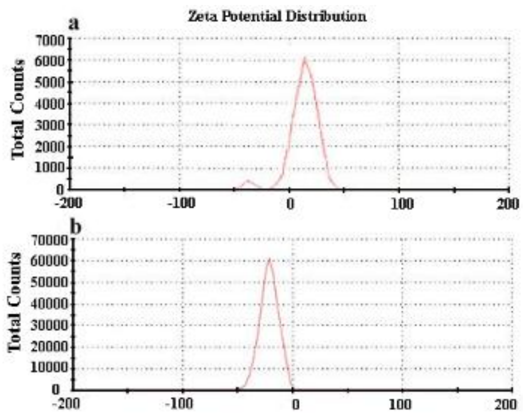

Figure S1 The Zeta patterns of (a) GO with CTAB and (b) Cu_3P nanoparticles

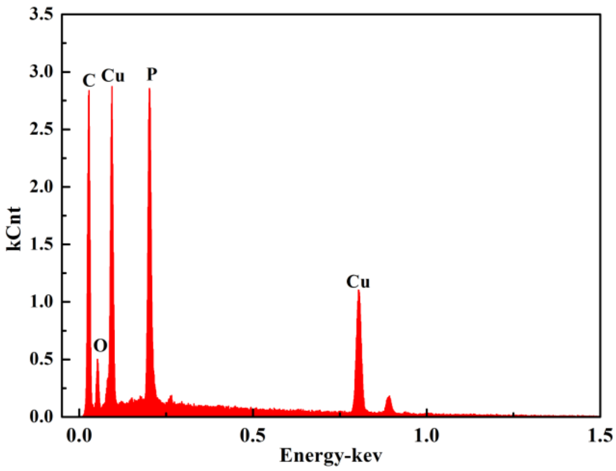

Figure S2 EDS of the as-prepared Cu_3P/RGO nanocomposite.

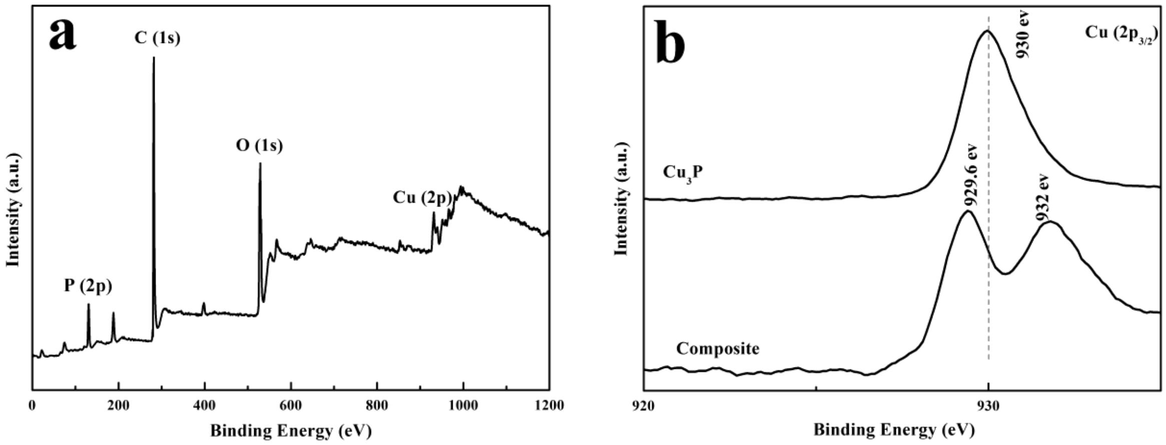

Figure S3 XPS spectra of (a) the survey scan, (b) Cu $2p_{3/2}$ in Cu_3P and Cu_3P/RGO

As shown in Fig. S3, the Cu $2p_{3/2}$ peaks of the composite were multiplet split peaks, representing different chemical environment of Cu. The contributions are discerned at 929.6 and 932 eV. The higher BE peak at 932 eV is assigned to the interaction of Cu(Ⅰ) and residual O on

the surface of RGO. The lower BE peak at 929.6 eV comes from Cu_3P of composite. But this Cu2p peak is shifted compared with that of Cu_3P, by about 0.4 eV, to a lower binding energy. This shift might be attributed to a partial electron transfer between Cu_3P and RGO, which is agreement with the result of Raman spectra.

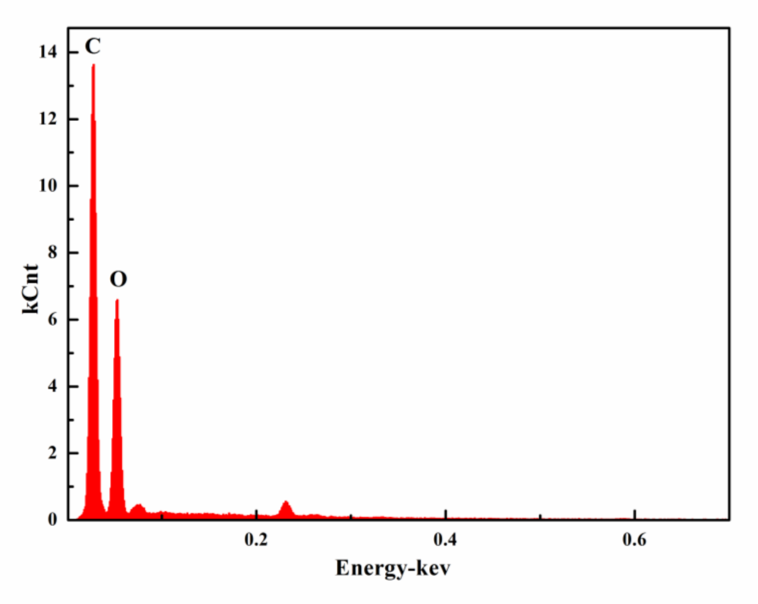

Figure S4 EDS of GO.

The integral area ratio of C and O peaks is about 1.32:1.

Cycle number	11	22	33	44	55	66
Density(mA/g)	100	200	400	800	1600	Back to 100
Reversible capacity of Cu_3P (mAh/g)	925	875	790	691	624	888
Reversible capacity of Cu_3P/RGO (mAh/g)	387	310	220	174	143	195

Table S1. Rate performances of the Cu_3P and Cu_3P/RGO nanocomposite.
